# The Relationships Between Career-Related Emotional Support From Parents and Teachers and Career Adaptability

**DOI:** 10.3389/fpsyg.2022.823333

**Published:** 2022-12-22

**Authors:** Yuan Song, Fang Mu, Jiahong Zhang, Mingchen Fu

**Affiliations:** ^1^Teaching Affairs Office, Fujian Normal University, Fuzhou, China; ^2^College of Education, Fujian Normal University, Fuzhou, China; ^3^Mental Health Education and Counseling Center, Sun Yet-sen University, Guangzhou, China; ^4^Institute of Moral Education, Nanjing Normal University, Nanjing, China

**Keywords:** parental support, teacher support, career adaptabilities, emotional support, male students at Normal Universities

## Abstract

Career-related support from parents and teachers plays an essential role in the process of promoting young people’s career development. This study examined the relationship between parents’ and teachers’ career-related support and career adaptability among Chinese male primary school preservice teachers (*N* = 772). The participants completed the Career Adapt-Abilities Scale–Short Version (CAAS), the Career-Related Parental Support Scale (CRPSS), and the Career-Related Teacher Support Scale (CRTSS). Results showed that a high level of emotional support from parents and teachers had more effects on the career adaptability of Chinese male primary school preservice teachers then other aspects in the scale, and teachers’ emotional support is more important than parents’ emotional support. Notably, there is a complex correlation among education level and occupation of parents and their children’s occupational adaptability. These findings carry implications for supporting teachers and parents in facilitating preservice teachers’ career adaptability. Future research could identify the differential effects of different forms of teacher support and parental support relate to career adaptability.

## Introduction

According to career construction theory (CCT) (Savickas, 1997; Savickas, 2005, 2013), career adaptability depicts the multiple psychological resources involved in the problem-solving process in one’s career development, including career concern, career control, career curiosity, and career confidence. Over the past decade, there has been a burgeoning interest in career adaptability (Hirschi, 2009; Savickas and Porfeli, 2012; Savickas, 2013; Guan et al., 2014; Tolentino et al., 2014). Such interest has been borne out of numerous pressures to adapt to the fast-devolving global labor market (Rousseau, 1995; Restubog et al., 2011) and the significance challenge of school-to-work transition for undergraduate students (Tian and Fan, 2014). To this end, career adaptability is a vital competency for Chinese students confronting uncertain career prospects (Koen et al., 2012).

In keeping with CCT, studies have suggested that the family and school environment are important contextual factor that shape the study-to-work transition (Hargrove et al., 2002; Restubog et al., 2010; Garcia et al., 2011). Students seek emotional support from parents and teachers for their personal growth and adaptation to their career (Turner et al., 2003). Having parental support provides resources necessary to enable career exploration, and the confidence and motivation to pursue their career goals (Nota et al., 2007; Howard et al., 2009; Restubog et al., 2010; Garcia et al., 2011; Ginevra et al., 2015; Guan et al., 2015). Thus, this article examines the role of emotional support from parents and teachers in influencing the effects of career adaptability. However, although prior studies have examined the significant relationships between career-related support from parents and teachers and career adaptability (Hirschi, 2009; Guan et al., 2016), current research on career-related support from parents and teachers in China is still fairly-limited. When the employment situation in the field of education in China is changing rapidly, it is considered especially significant to study this topic with reference to preservice teachers. Therefore, investigating this relationship would provide new insights into further explore professional development of primary school preservice teachers theoretically and provide corresponding policy advice practically.

### Career-Related Parental Support and Career Adaptability

Research conducted in the West has indicated that career-related parental support has an important, positive effect on several career development variables such as career self-efficacy (Turner and Lapan, 2002; Alliman-Brissett et al., 2004; Restubog et al., 2010), career identity (Dietrich and Kracke, 2009), career decision making (Simmons, 2008; Garcia et al., 2015; Retnam et al., 2018), career identity (Rogers et al., 2018), career expectation (McWhirter et al., 1998), career exploration (Dietrich and Kracke, 2009; Dietrich et al., 2011), career-related stress (Du and Xie, 2005) and career salience (Diemer, 2007). In conformity with CCT, as an important contextual factor, parent support tends to shape the study-to-work transition or career adaptability (Hargrove et al., 2002; Restubog et al., 2010; Garcia et al., 2011).

Notably, participants investigated in the majority of research on career-related parent support comprised from technical school and vocational school (Zhang et al., 2015, 2020), general high schools (Cheng and Yuen, 2012; Dietrich and Salmela-Aro, 2013), and Universities graduate (Restubog et al., 2010; Lee and Mun, 2011), as well as special populations such as African American high school students (Constantine et al., 2005; Gushue and Whitson, 2006), Korean Collegiate Student Athletes (Moon, 2017), Mexican American high school girls (McWhirter et al., 1998), and Chinese immigrant youths (Ma and Yeh, 2010).

Additionally, research in career-related parental support in China is a quite new phenomenon, especially when compared to Western countries. A study investigating 324 Chinese Medical University students revealed that the more career-related parent support students perceived, the less difficulties they experienced in their career decision-making (Blustein et al., 2002). Another study evaluating indicated career-related parental support have positive effect on Chinese University students’ career adaptability (Guan et al., 2016). Moreover, when it comes to the research on one-child and more-than-one-child families, there are significant differences between them in career related parental support. Furthermore, gender differences, as well as regional differences (rural and urban) in career-related parental support were also interestingly (Sun et al., 2015). However, given the significant cultural differences (e.g., parenting styles; expectations; and aspirations) between Eastern and Western countries, the findings from Western studies cannot simply be applied to Chinese, it is considered especially important to study this topic with reference to Chinese male primary school preservice teachers.

### Career-Related Teacher Support and Career Adaptability

Previous research have shown significant associations between teacher support and career adaptability variables such as goals and aspirations (Farmer, 1985; Howard et al., 2009), decision-making self-efficacy (Kenny et al., 2010; Perry et al., 2010), career commitment (McWhirter et al., 1998), career planning (Kenny et al., 2010; Perry et al., 2010; Di Fabio and Kenny, 2015), outcome expectations (Gushue and Whitson, 2006), persistence (Bonneville-Roussy et al., 2013), self-perceived employability, self-efficacy, career outcome expectations (Di Fabio and Kenny, 2015), career intention (Lai, 2010), career development self-efficacy, the amount of relevant career trajectory information they possessed (Cheung and Arnold, 2014), goal mastery and academic efficacy (Kenny et al., 2010). In addition, we can draw upon insights offered by CCT (Savickas, 2002) to inform our understanding of how teachers’ support help students to pursue their career goals.

Empirically, a number of qualitative studies revealed that teacher support was perceived to benefit students’ school-to-work transition, academic ambition and career decision-making aspirations (Midgley et al., 1989; Mortimer et al., 2002; Robb et al., 2007). For example, a study by Robb et al. (2007) was found out that students’ confidence, the desire to achieve and career decision making were all influenced by teachers, in which participants were 16-year old students mainly from socio-economically disadvantaged backgrounds. Specifically, it also has been seen that providing specific help when the students’ parents couldn’t also influence students’ career development (Robb et al., 2007). Moreover, given that there are different ways of understanding teacher support, it is not surprising that different theories have been developed to reflect this form of support. Social cognitive career theory (Lent et al., 1994, 2000; Lent, 2012) highlights the significance of the emotional support that teachers provided in the process of helping students improve career social adaptability (Lent et al., 1994, 2000; Lent, 2012).

While teacher support in general was perceived to have significant and positive influence on most aspects of career development, most of it focus on general teacher support to students for their educational and personal development rather than on specific support for career development. There was an obvious need to explore career-related teacher support from the perceptions of Chinese male primary school preservice teachers. Additionally, it is clearly there is need for much better follow-up support. For example, one study showed that a majority of students (65%) reported that career-related support ended after graduation (Packard et al., 2012). Moreover, there is a significant lack of relevant research in the Chinese literature, which urgently needs to be tailored to suit Chinese context and each student in reality.

### The Relationship Between Socioeconomic Status, Parental Support, and Career Adaptability

Families all differ in the extent to which they can offer support, due to family socioeconomic status (SES) (Fu and Yang, 2009; Yu, 2010; Wang et al., 2013). Traditionally, China is a country that attaches great importance to “family” and family values. Parents usually play a very important role in their children’s education and career development, and they usually possess high expectations regarding their children’s future (Hou and Leung, 2011; Hou et al., 2012; Liu et al., 2015). This can be traced in part to the Chinese cultural stance of ‘Wang Zi Cheng Long’, ‘Wang Nü Cheng Feng’ (望子成龙,望女成凤)–meaning parents hope that their children will have a bright future. Usually, families tend to invest great efforts in supporting a single child’s career path.

Studies suggest that students from high SES background are more likely to have those parents who are not only actively encourage exploration in relation to career paths, but also instrumental in helping with career adaptability. In contrast, those from low SES background typically do not receive any significant instrumental help from parents regarding career exploration or obtaining information about career-related opportunities (Blustein et al., 2002). However, even when parents from low SES cannot provide sufficient instrumental and financial assistance, they can still support their children’s career adaptability through positive emotional support and verbal encouragement. For all students, this type of encouragement from parents can strengthen career aspiration and self-efficacy.

### Research Gaps

Although extant studies have covered a number of variables related to career development, there still remains several gaps in the research field that are worth exploring. It would be helpful to practitioners to know more about how different forms of teacher support and parental support relate to career adaptability (Savickas, 2005, 2012). Which forms of support are most beneficial? And how does the emotional support of teacher and parent affect students’ career adaptability attitudes and strategies? Such kinds of questions may be answered in this research, through exploring the interrelationship between career-related teacher, parental support and career adaptability.

Our knowledge of the current situation of career-related support from parent and teacher for Chinese male primary school preservice teachers is inadequate. It can also be seen that there are several limitations need to be addressed in future Chinese research. First, the previous participants have been either University graduates (Hu, 2009; Sun et al., 2015) or high school students (Cheng and Yuen, 2012). Other types of participants need to explore in future research, such as male primary school pre-service teachers. Second, we have yet to explore relationships between career-related support from parent and teacher and career adaptability in Chinese contexts, especially in emotional support. As the majority of studies on support from teacher and parent in career adaptability were conducted in Western countries, future research on this topic may be well-targeted on issues related to a specific country and culture as well, such as Chinese Culture, differentiated according to the characteristics of each culture to suit the geographical demographics and work context. In some situations, for example where classes are very large and many teachers may work across different classes, providing individualized support to students is very much more difficult. Comparative studies could allow researchers to discover commonalities across cultures as well as identifying unique features of teacher support needed in each setting (Nilsson et al., 2007).

In summary, the issues described above have given rise to the execution of the following studies which aimed to explore and then conceptualize the career-related emotional support from parent and teacher, and to explore of theoretical concepts and the interaction effects of career-related teacher and parental support and career adaptability in Chinese male primary school preservice teacher population.

### Theoretical Framework

According to career construction theory, individuals shape an identity and build a career path over a period of time, largely through interacting with and observing others in various roles and situations, such as family and school (Savickas, 2012). It is reasonable to hypothesize that support and advice from parents and teachers is likely to play a salient role in career adaptability development in Chinese male primary school preservice teachers. The concept of career adaptability refers to “the readiness to cope with the predictable tasks of preparing for and participating in the work role and with the unpredictable adjustments prompted by changes in work and working conditions” (Savickas, 1997, p. 254). This concept includes four aspects—namely, concern (understanding that one must choose and prepare for a working life), control (having self-discipline in pursuing a chosen career path and the ability to make any necessary occupational transitions), curiosity (exploration of the fit between oneself and the working world), and confidence (feelings of self-efficacy concerning the ability to successfully execute and adhere to one’s educational and vocational choices).

In this study, we take the support of parents and teachers as independent variables, while the career adaptability of Chinese male primary school preservice teachers as dependent variables to study the relationship between them. Our hypothesis is consistent with results from prior studies, which demonstrated evidence of positive relationship between parental and teacher support and students’ career development (Fouad, 2007; Yuen, 2011; Joyce and Early, 2014; Guan et al., 2015, 2016). There is an extensive literature on this topic. Young (1994) indicated that parents are usually the primary influence in encouraging adolescent sons and daughters to explore their career targets and plan their appropriate paths. Similarly, theories such as the SCCT (Lent et al., 1994, 2000) suggest that teachers may provide career role models and skill development opportunities for students, thus enriching their learning experience. Therefore, there is a reason to argue that the support of parents and teachers might hold benefits among Chinese male primary school preservice teachers career adaptability as existing evidence.

### The Present Study

**FIGURE 1:**
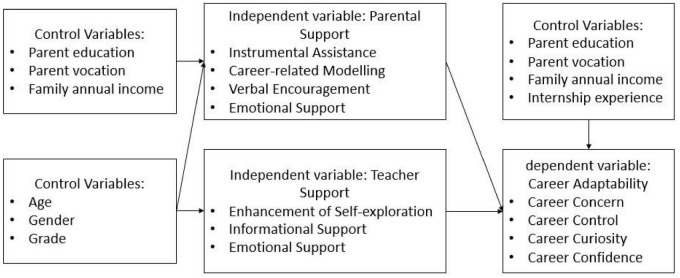
Theoretical framework.

This study aims to explore how teachers and parents could facilitate career adaptability of male students who majored in primary school education in a Normal University. Based on the above review of related research on SES, career-related parental support, career-related teacher support and students’ career adaptability, we proposed the theoretical framework of this study in Figure 1.

Specifically, we tested the following hypotheses in this research as follows:

Hypothesis 1: Career-related teacher support (i.e., enhancement of self-exploration, informational support, and emotional support) will predict career adaptability of male students at Normal Universities.
Hypothesis 2: Career-related parental support (i.e., instrumental assistance, career-related modeling, verbal encouragement, and emotional support) will predict career adaptability of male students at Normal Universities.

## Materials and Methods

### Participants

Initially 772 University male students majoring in primary education at a Normal University in a southeast province, China participated in the survey. We only invited male students to take part in this study because despite the participants in the previous studies came from a variety of different settings and age groups, but Chinese male primary school preservice teachers have been somewhat ignored by researchers in the past. A study in Chinese, for example, reported that the total score of career adaptability of boys was significantly higher than that of girls (Li et al., 2013). The reason may be that in contrast, fear of disappointing parents was positively associated with career exploration for boys but not for girls (Vignoli et al., 2005). And in today’s Chinese society, men bear greater pressure and more social expectations in social life, so they may take a more proactive attitude to explore and make better decisions. On the other hand, compared with girls, the experience of employment guidance shows that boys still have certain advantages in employment. More contact with the professional world and the potential advantages in this employment may be the reason why the total score of career adaptation of boys is higher than that of girls. Despite those evidence, there have been very few research that has examined in more detail gender differences in interrelationships among these variables of parental support and career adaptability. Therefore, this study aims to address this research gap and then provides researchers with a better understanding of the role of gender in this domain of students’ career adaptability. We removed data of 7 participants as they provided incomplete responses, and data of the rest 765 participants were used for further analyses of this study. Respondents’ ages ranged from 18 to 25 (mean age = 19.73 years, SD = 1.23).

The majority of students were sophomores (34.9%, *n* = 267), while there were 25.2% junior students (*n* = 193) and 24.4% freshmen (*n* = 187). Only 15.4% of the participants were seniors (*n* = 118). For the family annual income, over half of the sample students were from families with about annual income lower than $1,100 (RMB 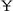
7000) and only 15.0% of the sample students were from family with income higher than $1,800 (RMB 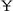
12,001). For the parent vocation, the majority of fathers (41.1%) and mothers (56.0%) were unskilled and semi-skilled workers. There were 30.5% of fathers and 21.6% of mothers were technicians. The minority of fathers (29.4%) and mothers (22.5%) were managers or at other senior staff positions. Data indicated that the majority of fathers (76.9%) and mothers (86.1%) had a secondary diploma or below (see Table 1 for details).

**TABLE 1:** Frequencies of the demographic information.

		*n*	%
Grade	Freshman	187	24.44
	Sophomore	267	34.90
	Junior	193	25.23
	Senior	118	15.42
Family annual income	$769 (RMB 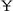 5000) or lES_PSs	192	25.10
	$770–$1077 (RMB 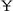 5001– 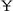 7000)	197	25.75
	$1078–$1538(RMB 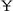 7001– 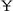 10,000)	160	20.92
	$1539–$1846(RMB 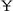 10,001– 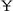 12,000)	101	13.20
	$1847–$3077(RMB 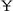 12,001– 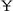 20,000)	64	8.37
	$3078–$4615(RMB 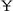 2001– 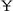 30,000)	35	4.58
	$4615 (RMB 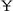 30,000) or more	16	2.09
Father vocation	Unskilled and semi-skilled worker	307	40.13
	Technician	233	30.46
	Ordinary civil servant	76	9.93
	Professional and technical personnel and middle-level administrative personnel	134	17.52
	Senior professional and senior executive	15	1.96
Mother vocation	Unskilled and semi-skilled worker	428	55.95
	Technician	165	21.57
	Ordinary civil servant	69	9.02
	Professional and technical personnel and middle-level administrative personnel	96	12.55
	Senior professional and senior executive	7	0.92
Father education	No formal education or primary	134	17.52
	Junior secondary	260	33.99
	Senior secondary or vocational secondary	194	25.36
	Associate degree	99	12.94
	Bachelor	69	9.02
	Master	5	0.65
	PhD	4	0.52
Mother education	No formal education or primary	269	35.16
	Junior secondary	246	32.16
	Senior secondary or vocational secondary	144	18.82
	Postsecondary	60	7.84
	Bachelor	43	5.62
	Master	3	0.39
Internship	PhD		86.14
	No	539	70.46
	Yes_	226	29.54
Total		765	100

### Measurements

#### Dependent Variables

In this study, we regard four aspects of career adaptability, namely, concern, control, curiosity, and confidence, as the dependent variables. The career adapt-abilities scale–short version (CAAS) (Savickas and Porfeli, 2012; Maggiori et al., 2015) was used to measure individuals’ adaptability in four dimensions (concern, control, curiosity, and confidence), with three items in each dimension. This version of the scale has sound evidence of reliability and validity in China (Zhang et al., 2021). It uses a 5-point Likert-type response mode with 5 = strongest and 1 = not strong. Higher total and sub-scale scores indicate greater adaptability. The Cronbach’s alphas of the four subscales ranged from 0.82 to 0.86.

#### Independent Variables

The four aspects of career-related parental support (i.e., instrumental assistance, career-related modeling, verbal encouragement, and emotional support) and the three types of teacher support (i.e., the enhancement of self-exploration, instrumental support and emotional support) were included in this study as independent variables.

The 24-item career-related parental support scale–Chinese version (Zhang et al., 2020) was used to assess students’ perceptions of career-related support they had received. They were required to consider this support from four perspectives—instrumental assistance, career-related modeling, verbal encouragement, and emotional support. It is a scale with 5-point Likert-type response with 1 strongly disagree and 5 strongly agree, and has proved to have good reliability and validity in mainland Chinese college students (Zhang et al., 2020). Higher scores on the instrument indicate stronger parental support. Cronbach’s alphas of subscales ranged from 0.88 to 0.91 in this study.

The 16-item career-related teacher support scale was originally developed to assess college students’ perceptions of career-related support they had received (Zhang et al., 2021). They were required to consider this support from three perspectives–the enhancement of self-exploration, instrumental support and emotional support. It is a scale with 5-point Likert-type response with 1 never and 5 always, and has proved to have good reliability and validity in mainland Chinese college students (Zhang et al., 2021). Higher scores on the instrument indicate stronger teacher support. Cronbach’s alphas of subscales ranged from 0.93 to 0.94 in this study.

#### Control Variables

The control variables included student basic information (i.e., gender, age, grade), student internship experience, and family socioeconomic status (i.e., family annual income, parental vocations, and parental education level). Specifically, internship experience is asked with a single question about whether they had participated in an internship, and the answers were coded as 1 = Yes and 0 = No. There were seven levels of family annual income, with 1 = $770 (RMB
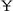
5000), 2 = $770–$1077 (RMB
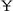
5001–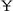
7000), 3 = $1078–$1538 (RMB
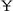
7001–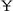
10,000), 4 = $1539–$1846 (RMB
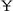
10,001–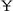
12,000), 5 = $1847–$3077 (RMB
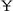
12,001–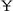
20,000), 6 = $3078–$4616(RMB
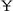
20001–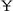
30,000) and 7 = $4615 (RMB 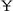
30,001) or more. There were five types of parent vocation provided, including 1 = unskilled and semi-skilled worker, 2 = technician, 3 = ordinary civil servant, 4 = professional and technical personnel and middle-level administrative personnel, and 5 = senior professional and senior executive. There were seven levels of the parent education, with choices of 1 = no formal education or primary, 2 = junior secondary, 3 = senior secondary or vocational secondary, 4 = associate degree, 5 = bachelor, 6 = master, and 7 = PhD.

### Procedure

We asked for students’ written consent before participating the survey. All participants were informed that (a) it was entirely voluntary to participate in this study; (b) they could withdraw at any time without consequences; (c) their answers would be confidential, and there is no right or wrong answer; and (d) their participation would be highly appreciated. Participants were requested to complete online surveys during class periods within 30 min.

### Data Analyses

#### Statistical Analyses of Survey Data

We conducted a two-step linear regression analysis to examine the amount of variance in the four aspects of career adaptability (concern, control, curiosity, and confidence) that can be explained by students’ basic information and career-related support from parents and teachers. For the four models of career adaptability, a block of student basic information was entered as control variables in model one, including age, grade, family income, parent educations, and vocations (which is a categorical variable), and internship experience. Then, support from parents and teachers were added as a second block of variables to the second model.

We reported the level of significance for each individual independent variable and the change in *R*^2^ created by the second block of variables. Finally, we conducted an *F* test to evaluate whether there was a significant improvement of the second model, compared with the first simpler model, after adding the variables of the parental and teacher support.

## Results

Table 2 showed the descriptive statistics and inter-correlations among the continuous variables that were included in the multiple regression analysis (i.e., career-related parental support, career-related teacher support and the four aspects of career adaptability). As shown in Table 2, all variables were positively and significantly associated with each other, with correlation coefficients ranging from 0.13 to 0.87. Therefore, our research results completely confirm hypothesis 1 and hypothesis 2. Specifically, there were low to medium levels of correlations between career-related parental support and career adaptability, with correlation coefficients ranging from 0.20 to 0.35. The correlation coefficients of the relationships between career-related teacher support and career adaptability were a little bit higher, ranging from 0.25 to 0.44. Additionally, there were medium to high levels of correlations of variables within the career-related parental support (*r* = 0.32–0.71), career-related teacher support (*r* = 0.70–0.77), and career adaptability (0.80–0.87).

**TABLE 2:** Correlations among key variables (*n* = 765).

	IA_PS	CM_PS	VE_PS	ES_PS	ESE_TS	IS_TS	ES_TS	Conc	Cont	Curi	Conf
Valid	765	765	765	765	765	765	765	765	765	765	765
Missing	0	0	0	0	0	0	0	0	0	0	0
Mean	3.33	3.87	4.24	3.94	3.15	3.37	3.47	3.29	3.40	3.36	3.403
SD	0.78	0.74	0.68	0.73	0.87	0.82	0.81	0.91	0.86	0.85	0.876
Minimum	1	1	1	1	1	1	1	1	1	1	1
Maximum	5	5	5	5	5	5	5	5	5	5	5
IA_PS	—										
CM_PS	0.50***	—									
VE_PS	0.32***	0.62***	—								
ES_PS	0.57***	0.63***	0.71***	—							
ESE_TS	0.38***	0.24***	0.13***	0.23***	—						
IS_TS	0.32***	0.28***	0.26***	0.27***	0.75***	—					
ES_TS	0.34***	0.3***	0.31***	0.34***	0.7***	0.77***	—				
Conc	0.25***	0.28***	0.27***	0.34***	0.31***	0.34***	0.43***	—			
Cont	0.21***	0.27***	0.29***	0.32***	0.25***	0.31***	0.41***	0.80***	—		
Curi	0.25***	0.28***	0.28***	0.34***	0.31***	0.33***	0.44***	0.84***	0.82***	—	
Conf	0.23***	0.27***	0.32***	0.35***	0.28***	0.31***	0.44***	0.84***	0.84***	0.87***	—

***p < 0.001; CM_PS, career-related modeling; Conc, concern; cont, control; conf, confidence; curi, curiosity; ES_PS, emotional support from parents; ES_TS, emotional support from teachers; ESE_TS, enhancement of self-exploration; IA_PS, instructional assistance; IS_TS, information support; PS, parental support; TS, teacher support; VE_PS, VE_PSrbal encouragement.

Table 3 showed results of linear regression analyses. With respect to career concern, age, grade, and some of the father’s vocation (i.e., technician, ordinary civil servant, and professional and technical personnel and middle-level administrative personnel [hereafter referred to as professional]) were significant control variables, but these variables only explained a very small percentage of the variation in concern, with adjusted *R*^2^ = 0.03. After adding a block of career-related variables to Model 2, variables that were significantly and positively associated with career concern included age (β = 0.09, *p* < 0.05), emotional support from parents (β = 0.23, *p* < 0.001) and emotional support from teachers (β = 0.40, *p* < 0.001). Additionally, the variables of grade (β = 0.11, *p* < 0.05) was negatively associated with concern. For parents’ vocations, results showed that comparing with those whose fathers worked as unskilled or semi-skilled workers, those whose fathers were technicians (β = 0.20, *p* < 0.01) and professionals (β = 0.30, *p* < 0.01) had higher career concern. In contrast, comparing with those whose mothers were unskilled or semi-skilled workers, those whose mothers worked as professionals (β = 0.27, *p* < 0.05) reported less career concern.

**TABLE 3:** Results of hierarchical regression analysis for career concern, control, curiosity, and confidence (*n* = 765).

		Concern		Control		Curiosity		Confidence	
					
		M1	M2	M1	M2	M1	M2	M1	M2
Age		0.12*	0.09*	0.09*	0.07	0.11*	0.08*	0.07	0.05
Grade		−0.14*	−0.11*	–0.10	–0.07	−0.11*	–0.07	–0.06	–0.03
Income		0.04	0.03	0.05*	0.04	0.06**	0.05*	0.05*	0.04*
Father education		–0.02	–0.04	–0.05	−0.07*	–0.03	–0.06	–0.03	–0.06
Mother education		0.04	0.03	0.07	0.07*	0.09*	0.09**	0.07	0.07*
Father vocation (reference group: Unskilled and semi-skilled worker)	Technician	0.25**	0.20**	0.16	0.11	0.19*	0.15*	0.11	0.07
	Ordinary civil servant	0.36**	0.23	0.26*	0.15	0.29*	0.18	0.25	0.14
	Professional^a^	0.36**	0.30**	0.29*	0.24*	0.28*	0.22*	0.26*	0.2
	Senior^b^	0.11	–0.08	0.20	0.01	0.26	0.08	0.16	–0.06
Mother vocation (reference group: Unskilled and semi-skilled worker)	Technician	–0.08	–0.11	–0.01	–0.04	–0.06	–0.09	–0.01	–0.04
	Ordinary civil servant	–0.24	–0.23	−0.33*	−0.32**	−0.28*	−0.28*	–0.24	–0.22
	Professional^a^	–0.19	−0.27*	–0.10	–0.17	–0.2	−0.27*	–0.11	–0.18
	Senior^b^	–0.16	–0.23	–0.21	–0.26	–0.37	–0.42	0.14	0.1
Internship		0.11	0.06	0.1	0.06	0.05	0	0.13	0.08
IA_PS			–0.01		–0.04		–0.03		–0.02
CM_PS			0.06		0.04		0.06		0.01
VE_PS			–0.01		0.05		0.02		0.11
ES_PS			0.23***		0.19**		0.19**		0.18**
ESE_TS			0.00		–0.07		0.04		–0.02
IS_TS			0.01		0.001		–0.07		–0.07
ES_TS			0.40***		0.41***		0.41***		0.46***
Adjusted *R*^2^		0.03	0.24	0.02	0.22	0.03	0.25	0.03	0.25
Δ Adjusted *R*^2^			0.21						
*F*-value		2.48**	12.37***	2.4**	11.29***	2.98	13.03	2.45	12.91
Δ F (M1 vs. M2)			30.76***		187.6***		216.31***		

*p < 0.05; **p < 0.01; ***p < 0.001.

*^a^Professional and technical personnel and middle-level administrative personnel.*

*^b^Senior professional and senior executive. PS, career-related modeling; Conc, concern; cont, control; conf, confidence; curi, curiosity; ES_PS, emotional support from parents; ES_TS, emotional support from teachers; ESE_TS, enhancement of self-exploration; IA_PS, instructional assistance; IS_TS, information support; PS, parental support; TS, teacher support; VE_PS, VE_PSrbal encouragement.*

Model 2 explains 21% more of the variation in concern (adjusted *R*^2^ = 0.24). Overall, after adding variables of the career-related parental and teacher support in Model 2, the concern model was significantly improved, with *F* = 30.76, *p* < 0.001.

With respect to career control, age, family annual income and two types of father’s vocations (i.e., ordinary civil servant and professional) were positively associated with control. In contrast, mothers who worked as ordinary civil servant were negatively associated with control. However, the control variables only explained 2% of the variation in career control (adjusted *R*^2^ = 0.02), which is very small. In Model 2, variables that were significantly and positively associated with career control included emotional support from parents (β = 0.19, *p* < 0.01) and emotional support from teachers (β = 0.41, *p* < 0.001). Moreover, for control variables, mother education (β = 0.07, *p* < 0.05) was positively associated with career control, whereas father education (β = -0.07, *p* < 0.05) was negatively related to career control. Moreover, comparing with those whose fathers worked as unskilled or semi-skilled workers, those whose fathers were professional (β = 0.24, *p* < 0.05) reported higher career control. And comparing with those whose mothers worked as unskilled or semi-skilled workers, those whose mothers worked as ordinary civil servant (β = -0.32, *p* < 0.01) rated a lower level of career control. Model 2 explains 20% more of the variation in satisfaction (adjusted *R*^2^ = 0.22). Overall, after adding variables of the career-related parental and teacher support in Model 2, the control model was significantly improved, with *F* = 187.6, *p* < 0.001.

For career curiosity of Model 1, age, family annual income, mother education and three types of father vocation (i.e., technician, ordinary civil servant, and professional) and one type of mother vocation (i.e., ordinary civil servant) were significant control variables, but they only explained 3% of the variation in curiosity (adjusted *R*^2^ = 0.03), which is very small. In Model 2, variables that were significantly and positively associated with career curiosity included age (β = 0.08, *p* < 0.05), family income (β = 0.06, *p* < 0.01), mother education (β = 0.09, *p* < 0.01), emotional support from parents (β = 0.19, *p* < 0.01), and emotional support from teachers (β = 0.41, *p* < 0.001). Additionally, comparing with those whose fathers worked as unskilled or semi-skilled workers, those whose fathers were technicians (β = 0.15, *p* < 0.05) and professionals (β = 0.22, *p* < 0.05) reported higher level of career curiosity. In contrast, comparing with those whose mothers worked as unskilled or semi-skilled workers, those whose mothers worked as ordinary civil servant (β = -0.28, *p* < 0.05) and professionals (β = -0.27, *p* < 0.05) had lower level of curiosity. Model 2 explains 22% more of the variation in satisfaction (adjusted *R*^2^ = 0.25). Overall, after adding variables of the career-related parental and teacher support in Model 2, the curiosity model was significantly improved, with *F* = 28.12, *p* < 0.001.

For career confidence of Model 1, family annual income, father’s who worked as professional were significant control variables, but they only explained 3% of the variation in confidence (adjusted *R*^2^ = 0.03), which was very small. In Model 2, variables that were significantly and positively associated with career confidence included emotional support from parents (β = 0.18, *p* < 0.01) and emotional support from teachers (β = 0.46, *p* < 0.001), family income (β = 0.04, *p* < 0.05), and mother education (β = 0.07, *p* < 0.05). Model 2 explains 22% more of the variation in satisfaction (adjusted *R*^2^ = 0.25). Overall, after adding variables of the career-related parental and teacher support in Model 2, the curiosity model was significantly improved, with *F* = 223.65, *p* < 0.001.

These results suggest that both emotional support from parents and teachers are the very significant and essential variables that are related to the four aspects of career adaptability. And notably, the coefficients of emotional support from teachers are larger than those of emotional support from parents. We provided detailed discussion and implications of these findings in Discussion section.

## Discussion

We examined relationships among career-related parental support, career-related teacher support, and career adaptability in the survey of Chinese male primary school preservice teachers. The CAAS was used to measure individuals’ adaptability while the 16-item Career-related Teacher Support Scale and the 24-item Career-related Parental Support Scale–Chinese Version were developed to assess college students’ perceptions of career-related support they had received. This study found that emotional support from parents and teachers have a significant relationship with career concern, career control, career curiosity and career confidence (Guan et al., 2015), and teachers’ emotional support is more important than parents’ emotional support. Notably, parents’ educational level and vocation also have an important relationship with career adaptability, which is more complex. Specifically, for the variable of education level, mother showed positive correlation while father showed negative correlation. On the contrary, when it comes to career, the mother is negatively correlated while the father is positively correlated. This completely supports hypothesis 1 and hypothesis 2. Therefore, this section will discuss these findings and their implications.

Specifically, this study found that compared with the other three sources of parental support (e.g., instructional assistance, career-related modeling, verbal encouragement) in the scale, emotional support has a more positive impact on children’s career adaptation. Although there is no specific study on the relationship between parents’ emotional support and children’s occupational adaptability, to some extent, these findings are consistent with prior research suggesting that emotional support from parents can promote children’s career development (Hirschi, 2009; Guan et al., 2016). Career-related support from parents has been widely recognized as valuable for adolescents and young adults (Farmer, 1985; Turner et al., 2003; Ali and Saunders, 2006; Leung et al., 2011; Ginevra et al., 2015; Guan et al., 2015), among which emotional support is a universal type. We found that parents’ instrumental support, career-related modeling, verbal encouragement and emotional support represent distinct dimensions of support provision. Crucially, emotional support, but not the other three support, consistently have a positive impact on the recipients (Wentzel et al., 2016). Additionally, socio economic status of the family influences mental health of student’s that builds up occupational aspirations (Gjerustad and von Soest, 2012) impacting overall psychological well-being of student’s. Guided by the self-determination theory, emotional support can stimulate children’s internal motivation for career adaptation. Good career related emotional support provided by parents can promote children’s career adaptability and alleviate the disadvantage of family economic status.

In addition, tradition limits parents support of instrumental support, career related modeling support, and verbal encouragement to some extent. That is, high traditionality impedes the enhancement of career adaptability. In China, for example, parents traditionally play a very significant role in influencing career aspirations of their children, usually hoping that their son or daughter will choose employment that takes them above the family’s current socioeconomic status (Liu et al., 2015; Powers and Myers, 2017). Therefore, parents don’t want to give their children too much information about their career. In so doing this research identifies emotional support from parents as an important boundary condition that determine the extent to which parental support can foster career adaptability.

Interestingly, the same as parental support, teachers’ emotional support obviously has a more positive impact on students’ professional adaptability than the other two factors (e.g., enhancement of self-exploration, information support, emotional support) in the scale. This can also be verified in previous studies. For example, Malecki et al. (2000) were influenced by Tardy’s (1985) “social support model,” and they defined teacher support as a set of purposeful strategies and actions that may be interpreted as instrumental, emotional, informational or appraisal. Additionally, Hamre and Pianta (2007) also emphasized instrumental and emotional support (and supportive classroom organization) as criteria for encouraging effective teacher-student interactions in the classroom (Hamre and Pianta, 2007). In general, given that many college students leave their parents for a long time for the first time when they going to college, so teachers become the ideal candidate for their emotional sustenance. Teachers commonly exert their influence on students by investing time, energy and enthusiasm into taking charge of their students’ personal lives, academic lives and career development. This in turn builds stronger connections between teacher and students, with added benefits in terms of emotional support (Shang, 2004; He, 2010; Jia, 2011). Teacher support has been found to have a unique effect on positive expectations regarding a chosen career path (Gushue and Whitson, 2006). It is reported that it also has a larger effect than parental support on students’ career-related decision making, and career planning (Kenny and Bledsoe, 2005; Cheung and Arnold, 2014), especially students from families with low socio-economic status. Moreover, when teachers’ emotional support stimulates students’ self-improvement, enhancement of self-exploration and information support will play a corresponding role better.

Notably, significant relationships were found among career concern, career control, career curiosity career confidence, and parental vocation—specifically, the positive effect of parents’ vocational types on career adaptability is enhanced by the father’s higher vocation, and weakened by the mother’s higher vocation. In other words, children of families whose fathers have better vocations have better career adaptability. This finding can be reflected in previous studies. Occupation is an indicator of socio economic status (Ganzeboom et al., 1992). In Chinese tradition, the father plays the role of making money to support the family, so the father’s high occupation represents the high economic status of the family. Socioeconomic status (SES) can also affect individuals’ personal resources (cognitive, affective, behavioral) and how they utilize and regulate these resources (Moffitt et al., 2001; Frank, 2004; Raffaelli et al., 2005). Parents’ vocational status and family wealth show that higher family economic status will promote their children’s education, and then promote their career adaptability (Carpenter et al., 1998; McMillan and Western, 2000; Gürel, 2011). When it comes to the mother’s career, on the one hand, in the tradition, the mother takes more responsibility for raising children, and the working time will occupy the time of raising children, resulting in too little mother’s intervention in the child’s career, so the mother’s career is negatively correlated with the child’s career adaptability. On the other hand, some highly professional mothers spoil their children with the help of their professional advantages and excessively interfere with their children’s career choices, resulting in their children’s lack of ability to adapt to their careers. Indeed, parental support is also vital for providing guidance on how to formulate career specific goals and to make educational and vocational choices that support those goals (Hargrove et al., 2002). In fact, it is an established practice in China that parents invest in their children’s education and training (Soresi et al., 2014). However, too much or too little interference will cause adverse consequences, which is difficult to cultivate children’s appropriate professional adaptability.

We also found that the mother’s education level was positively correlated with career curiosity, career control, and career confidence, while the father was negatively correlated. And compared with the father, the mother’s education has a greater impact on career adaptability. This is consistent with the previous research, parents’ level of education was positively related to adaptability (Martin et al., 2012). Shan (2019) conducted a study showed that the educational level of fathers is different, and the parenting styles of families may be no different. However, the degree of maternal education is statistically significant. In Chinese tradition, mothers undertake the important task of educating their children. Therefore, it may be that mothers who have longer and broader educational experience are able to more specifically engender greater cognitive-behavioral and affective resources to the many challenges adolescents experience through in-school and out-of-school life (Mansour and Martin, 2009). Receiving higher education is conducive to promoting the mother’s cultivation of children, so the higher the mother’s education, the higher the child’s professional adaptability.

## Conclusion

Career-related support from parent and teacher plays an essential role in the process of promoting male primary school preservice teachers’ career adaptability. Research has confirmed that emotional support of parent and teacher is positively associated with career adaptability among these factors. Career-related support from teachers is of particular importance for male primary school preservice teachers, especially those coming from families with low SES standing. And parents’ career and education are closely related to their children’s career adaptability. However, compared with research on career-related support from parent and teacher in Western countries, studies in China are still at a very preliminary stage. To date, there is only limited research data involving emotional support from parents and teachers to Chinese male primary school preservice teachers, and there is the potential for much more to be done to help students in this regard. Our results also have important practical implications. First, students, parents, and educators need to be aware that the support from parents and teachers can potentially promote the development of students’ career decision-making self-efficacy and career adaptability. However, parents and teachers also need to take into account the possible effects of the career-related support students may or may not be receiving from them. In some cases this support may be providing him or her with clear goals, emotional and material support, and a sense of direction. In other cases, it may be that the expectations of parents or teachers are unrealistic, and cause the student to experience ongoing stress and make bad choices. In the case of families where parental support is lacking or insufficient, teachers may need to take on more responsibility for supporting students, while at the same time helping their parents, where necessary, to seek more resources and social supports in the community. We hope that the research presented here sheds some light on the role of the support from parents and teachers and call for continued research on career adaptability.

## Limitation and Future Research Direction

The results of our study should be interpreted with caution in light of a number of limitations. First, the operationalization of emotional support in this study may limit the generalizability of the results, given that Chinese context may not be representative in other cultures. The findings should be for the sample was limited to male primary school preservice teachers in a Normal University in South China, which cannot generalize professional development of all the primary school preservice teachers. Hence, there is a need to include preservice teachers with a relatively larger range of educational backgrounds in future studies. Second, we used the CRPSS and CTPSS to explain the role of emotional support in promoting career adaptability. As such, we did not explicitly assess specific psychological needs such as competence and autonomy. Future research should examine the extent to which emotional support could promote autonomy and competence which may result in increased career adaptability.

## Data Availability Statement

The raw data supporting the conclusions of this article will be made available by the authors, without undue reservation.

## Ethics Statement

Ethical review and approval was not required for the study on human participants in accordance with the local legislation and institutional requirements. Participants provided written informed consent.

## Author Contributions

YS and JZ substantially contributed to the conception and design of the work, the analysis and interpretation of data for the work, and drafting the work or revising it critically for important intellectual content. FM drafted part of the manuscript. MF assisted the interpretation of data for the work, and provided final approval of the version to be published. All authors contributed to the article and approved the submitted version.

## Conflict of Interest

The authors declare that the research was conducted in the absence of any commercial or financial relationships that could be construed as a potential conflict of interest.

## Publisher’s Note

All claims expressed in this article are solely those of the authors and do not necessarily represent those of their affiliated organizations, or those of the publisher, the editors and the reviewers. Any product that may be evaluated in this article, or claim that may be made by its manufacturer, is not guaranteed or endorsed by the publisher.
